# Intermittent Parathyroid Hormone Accelerates Stress Fracture Healing More Effectively Following Cessation of Bisphosphonate Treatment

**DOI:** 10.1002/jbm4.10387

**Published:** 2020-08-06

**Authors:** Mahmoud M Bakr, Wendy L Kelly, Athena R Brunt, Bradley C Paterson, Helen M Massa, Nigel A Morrison, Mark R Forwood

**Affiliations:** ^1^ School of Medical Sciences and Menzies Health Institute Queensland Griffith University Gold Coast Queensland Australia; ^2^ School of Dentistry and Oral Health Griffith University Gold Coast Queensland Australia

**Keywords:** BISPHOSPHONATES, STRESS FRACTURE, BONE REMODELING, HEALING, PARATHYROID HORMONE, ULNA

## Abstract

Parathyroid hormone (PTH) and bisphosphonates (BPs), including alendronate (ALN), have opposing effects on bone dynamics. The extent to which PTH remains effective in the treatment of stress fracture (SFx) in the presence of an ongoing BP treatment has not been tested. SFx was induced in 150 female Wistar rats, divided into five equal groups (*n* = 30). All rats were pretreated with ALN (1 μg/kg^−1^/day^−1^) for 14 days prior to SFx induction, followed by ALN cessation or continuation for the duration of the experiment; this was combined with daily PTH (8 μg/100 g^−1^/day^−1^) on SFx induction for 14 days, followed by cessation or continuation of ALN after SFx induction or an equivalent vehicle as a control. Ulnas were examined 2 weeks or 6 weeks following SFx. Two toluidine blue‐ and two tartrate‐resistant acid phosphatase‐stained sections were examined for histomorphometric analysis using Osteomeasure software. There was a significant interaction between the effects of time and treatment type on the woven bone width and apposition rate, as well as an improvement in the woven bone architecture. However, woven bone variables remained unaffected by the cessation or continuation of ALN. Cessation of ALN increased osteoclast number when compared with the ALN‐PTH continuation group (*p* = 0.006), and vehicle (*p* = 0.024) after 2 weeks. There was a significant interaction between the effects of time and treatment type on the number of osteoclasts per unit BMU area and length. The number of osteoclasts per unit BMU area and length was significantly greater in ALN cessation groups. It was concluded that intermittent short‐duration iPTH treatment effectively increased remodeling of SFx with a concurrent BP treatment, provided that BP was ceased at the time of SFx. Our results could help develop shorter iPTH treatment protocols for the clinical management of SFxs and guide clinical decision‐making to cease BP treatment in cases of SFx. © 2020 The Authors. *JBMR Plus* published by Wiley Periodicals LLC. on behalf of American Society for Bone and Mineral Research.

## Introduction

Stress fractures (SFxs) account for about 1% to 7% of all athletic injuries.^(^ ^1^, ^2^ ^)^ The main difference between SFxs and acute fractures is related to the nature of loading of the bones. An acute fracture typically occurs because of a single maximal loading, whereas SFxs occur because of repetitive submaximal loading.^(^ ^1^ ^)^ Bisphosphonates (BPs) are stable analogues of pyrophosphatase. They are deposited on bone surfaces within minutes or hours of uptake. The mode of action on the osteoclast is radically different between non‐nitrogen‐containing BPs (first generation) and nitrogen‐containing BPs (second and third generation). Apoptosis is induced by non‐nitrogen‐containing BPs through the formation of a toxic analogue of adenosine triphosphate, whereas nitrogen‐containing BPs (second and third generation) target the enzyme farnesyl diphosphate synthase needed for posttranslational modification of small guanosine triphosphate‐ (GTP‐) binding proteins required for osteoclastic function.^(^ ^3^, ^4^ ^)^ All BPs have an affinity for bone tissue, more specifically to osteoclasts, because the acidic pH of resorption lacunae causes an intracellular uptake of BPs, leading to the internalization of substantial amounts of BP. Regardless of the inhibitory pathway, all BPs depress osteoclast activation and resorption.^(^ ^4^ ^)^

BPs are highly effective in the treatment of osteoporosis. Numerous large clinical trials demonstrate their efficacy in reducing bone turnover, increasing BMD, and reducing vertebral and nonvertebral fracture risk in patients with osteoporosis.^(^ ^5^, ^6^ ^)^ They are incorporated into the bone matrix and have a biological half‐life of more than 10 years.^(^ ^7^ ^)^ As the duration of antiresorptive treatments increased to 10 years or more, debate arose concerning the risks versus benefits of antiresorptive treatment, and the consequences of “frozen bone.”^(^ ^8^ ^)^ One of the side‐effects that emerged was a SFx in the proximal femoral diaphysis, which progressed to an atypical femoral fracture (AFF).^(^ ^8^, ^9^, ^10^, ^11^, ^12^, ^13^ ^)^ Recently, a case of fracture in the proximal ulna was also reported after prolonged BP treatment.^(^ ^14^ ^)^

It is now well‐accepted that the pathogenesis of AFF is its initiation as a SFx caused by an accumulation of unrepaired microdamage following suppression of bone turnover by BPs.^(^ ^15^, ^16^ ^)^ It is also acknowledged that initiation of the microdamage is facilitated by bone hypermineralization and accumulation of advanced glycation end‐products that reduce the toughness of the bone matrix.^(^ ^17^, ^18^, ^19^, ^20^ ^)^ This hypothesis is supported by the location of the AFF in areas of stress (lateral femur), and the fact that such fractures develop over a long period and are preceded by episodes of prodromal pain.^(^ ^15^ ^)^ There are a lot of similarities between SFxs and AFFs in terms of etiology^(^ ^15^, ^16^ ^)^ and possible management protocols.^(^ ^21^, ^22^, ^23^ ^)^ Therefore, we hypothesized that anabolic PTH treatment would accelerate SFxs, even in the presence of BP treatment.

Many studies confirm the anabolic effect of PTH and its correlation with the timing and duration of treatment.^(^ ^24^, ^25^ ^)^ Intermittent administration of PTH results in increased bone formation, and cortical bone volume and width. It also improves bone architecture and mechanical properties in different species.^(^ ^26^, ^27^, ^28^ ^)^ The potential for PTH to treat localized osseous defects and accelerate SFx healing has also been investigated.^(^ ^29^, ^30^, ^31^ ^)^ Conversely, combination therapy of PTH plus BP (alendronate [ALN]) impairs the anabolic function of PTH and its ability to enhance BMD.^(^ ^32^, ^33^ ^)^ For example, PTH treatment for 2 months after an antiresorptive agent increased bone formation and the mineralization rate, but it was less than that achieved with PTH treatment alone,^(^ ^34^, ^35^, ^36^ ^)^ though this observation has not been universal.^(^ ^37^ ^)^ So the question remains as to what extent PTH can remain effective in the presence of BP treatment.

We previously showed that BP treatment (risedronate) impaired healing of a SFx by reducing the volume of bone resorbed and replaced during remodeling.^(^ ^38^ ^)^ However, formation of a periosteal callus was not adversely affected. This woven bone reaction acts to return the bone to its original strength 2 weeks after SFx induction.^(^ ^39^, ^40^ ^)^ However, there is little analysis available for the combined effects of a BP with PTH. In the ulna SFx model, PTH increased BMC significantly by 7% at 4 weeks and BMD and BMC significantly by 10% and 7% at 8 weeks compared with controls, whereas ALN did not change BMD or BMC.^(^ ^41^ ^)^ PTH significantly stimulated bone formation by 114% at 2 weeks, increased intracortical resorption area by 23% at 4 weeks, and enhanced the ultimate force of the affected ulnas by 15% at 8 weeks. Similar to Kidd and colleagues,^(^ ^38^ ^)^ ALN significantly suppressed the bone formation rate by 44% compared with the control at 4 weeks.^(^ ^41^ ^)^ These data suggest that PTH could accelerate SFx remodeling, but the combined effects of PTH with BPs remain unknown.

The ulnar loading model in rats provides an effective approach to examine focal SFx remodeling with a known time course and precise anatomical location.^(^ ^22^, ^31^, ^38^, ^42^ ^)^ Patients receiving long‐term BP treatment may be at higher risk for development of SFx, and more serious complications including AFF.^(^ ^8^, ^9^, ^10^, ^11^, ^12^, ^13^ ^)^ Although some studies have investigated the effect of combined PTH‐BP therapy in osteoporosis, this is not the case for SFx and potential AFF.^(^ ^43^ ^)^ Therefore, the aim of the current experiment was to investigate the efficacy of a daily iPTH treatment for 14 days in acceleration of SFx remodeling and healing indices in the rat ulna in the presence or absence of a concurrent BP treatment (ALN).

## Materials and Methods

The Griffith University Animal Ethics Committee (Nathan, Queensland, Australia ) approved the experimental protocols (GU Ref No: MSC/02/13/AEC).

We induced an ulnar SFx in 150 female Wistar rats, 12 weeks of age, weight (300 ± 15 g). Rats were anesthetized with halothane and oxygen for loading. SFx was achieved in a single loading session of the forearm in axial compression at 2 Hz until an increase in displacement of 10% was reached, on average after approximately 8000 cycles or about 60 minutes (range, 4000 to 20,000). This produced a remarkably standardized SFx.^(^ ^22^, ^31^ ^)^ Accounting for variability, anesthesia, and recovery, four to five rats were loaded/day. Loading was performed in a loading device using a Linear Voltage Displacement Transducer (LVDT) to monitor displacement in the limb. Because of the natural ulnar curvature, axial compression was converted into bending forces with the lateral cortex in tension and the medial in compression.^(^ ^44^ ^)^ Loading involved cyclic compressive loading at 18 to 20 N load and 2‐Hz cycle frequency. Loading was stopped at a predetermined point when increased displacement reached 10%. As noted above, this level of stiffness loss consistently produced SFxs. A single injection of opioid analgesia (buprenorphine 0.05 mg/kg s.c.) was used following loading sessions.

Rats were divided into ALN‐, combined therapy‐ (ALN‐PTH‐), and vehicle‐ (VEH) treated groups (*n* = 15 each). PTH‐treated groups received s.c. injections of human PTH‐(1–34) peptide (Sigma‐Aldrich, St. Louis, MO, USA) dissolved in 0.9% saline with 1% rat heat‐inactivated serum in a final volume of 200 μL with a dose of 8 μg/100 g/day. PTH treatment was started 24 hours after SFx loading and continued daily for 14 days. ALN‐treated groups received s.c. injections of ALN in saline carrier, once daily at 1.0 μg/kg/day. The ALN treatment started 14 days before the SFx loading. The ALN dose was adjusted for the metabolic rate of the rat and equivalent to the human clinical dose.^(^ ^22^ ^)^ VEH groups received an equivalent dose of saline for each ALN injection and an equivalent dose of rat serum for each PTH injection in the corresponding treatment groups. Rats were euthanized at 2‐ and 6‐weeks post‐SFx (Table 1).

**Table 1:** Experimental Design of the Study

Group	14 days (post‐SFx)	6 weeks (post‐SFx)
Alendronate continuation (ALN_1_)	15	15
Alendronate cessation (ALN_2_)	15	15
Combined treatment with alendronate continuation (ALN‐PTH_1_)	15	15
Combined treatment with alendronate cessation (ALN‐PTH_2_)	15	15
Vehicle (VEH)	15	15

ALN_1_ = Pretreatment with ALN for 14 days before SFx, followed by daily ALN injections up to 6 weeks post‐SFx; ALN_2_ = pretreatment with ALN for 14 days before SFx, followed by cessation of ALN at time of SFx, no treatment up to 6 weeks; ALN‐PTH_1_ = pretreatment with ALN for 14 days before SFx, followed by daily ALN injections up to 6 weeks post‐SFx + 14 days of PTH treatment post‐SFx; ALN‐PTH_2_ = pretreatment with ALN for 14 days before SFx, followed by cessation of ALN at time of SFx + 14 days of PTH treatment post SFx; SFx = stress fracture; VEH = control for ALN‐PTH groups.

### Histomorphometry

**Fig 1:**
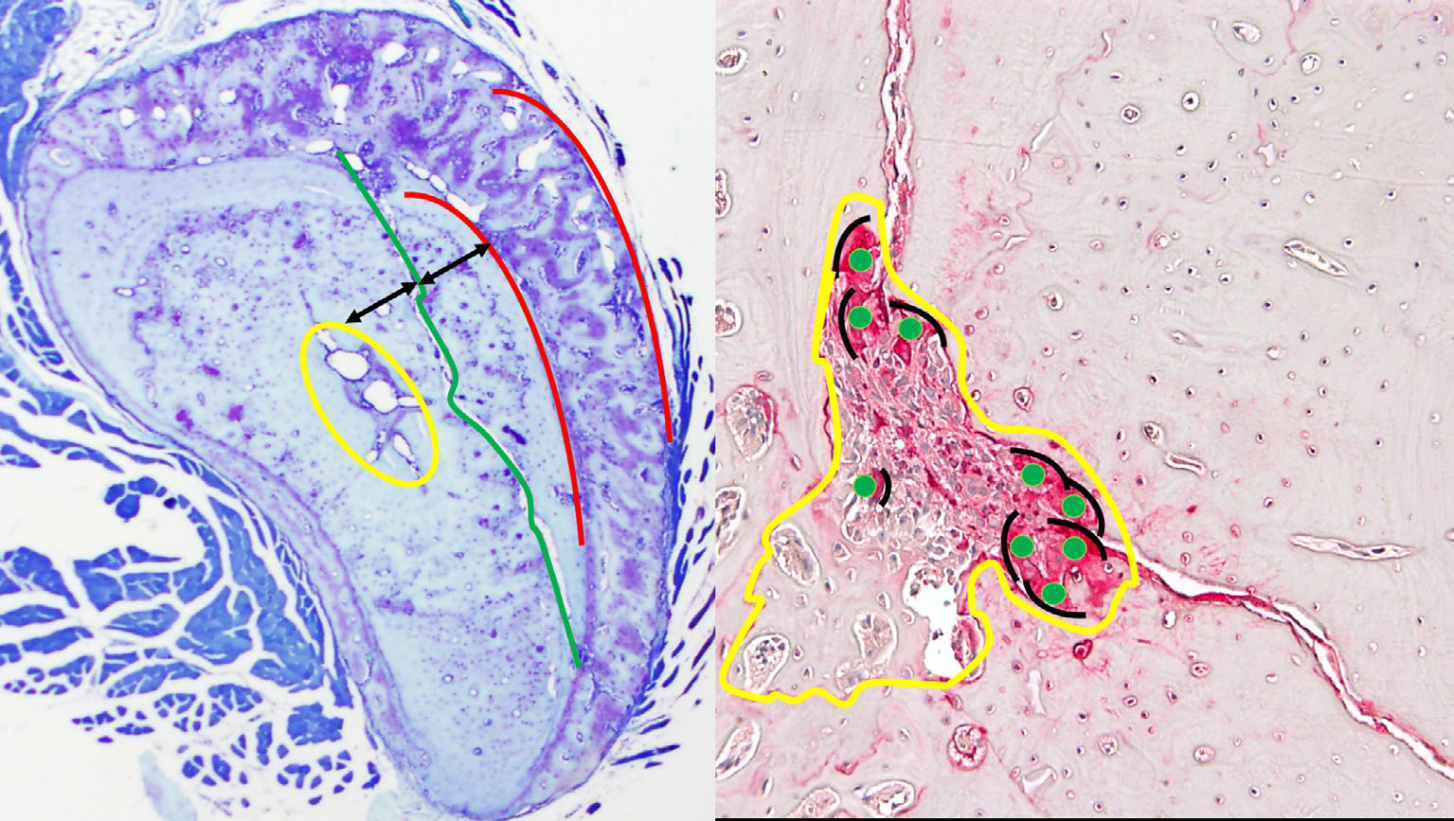
Left panel: Photomicrograph of a transverse section of a rat's ulna showing the standardized position chosen for histomorphometric analysis in this study along the stress fracture. To avoid any bias the slides selected for analysis were chosen at a position where the stress fracture (green) was midway between the outer cortical margins (red) and the inner medullary cavity (yellow; toluidine blue 2X). Right panel: A schematic diagram on a tartrate‐resistant acid phosphatase‐stained slide showing the boundaries of the BMU (yellow) with osteoclast count (green) and osteoclast perimeter (black; tartrate‐resistant acid phosphatase 10X).

Two toluidine blue and two tartrate‐resistant acid phosphatase‐stained sections were examined for histomorphometric analysis using Osteomeasure software (OsteoMetrics, Decatur, GA, USA). As previously described, histomorphometry was performed at a standard level along the SFx (Fig. 1*A*), where the microcrack was halfway between the medial cortical margin and the medullary cavity of the bone in transverse section.^(^ ^22^, ^31^ ^)^ The area of a BMU was defined as the total area that had been resorbed. Within this BMU, the area filled with new bone formation was defined as the healed area. A distinct cement line around a previously resorbed area of bone defined the healed area. The area that had been resorbed, but not yet filled by new bone formation, was defined as porosity. Morphometric measures included standard variables (Fig. 1):

1. Cortical bone area (Ct.Ar [mm^2^])
2. Cortical bone perimeter (mm)
3. Woven bone area (mm^2^)
4. Woven bone perimeter (mm)
5. Woven bone width (Wo.B.Wi [mm])
6. Length of SFx (μm)
7. Length of remodeling unit along the microcrack (μm)
8. Porosity BMU area (μm^2^)
9. Porosity BMU area perimeter (μm)
10. Erosion (unhealed) area (μm^2^)
11. Erosion (unhealed) perimeter (μm)
12. Healing area (μm^2^)
13. Healing area perimeter (μm)
14. Number of osteoclasts
15. Osteoclasts surface perimeter (μm)

From the standard variables measured above, the following derived variables were obtained:

1. Number of osteoclasts per μm^2^ of BMU area (number of osteoclasts /μm^2^)
2. Number of osteoclasts per μm of BMU length (number of osteoclasts/μm)
3. Healing percentage (healing area [μm^2^]/porosity BMU area × 100 [μm^2^])
4. Woven bone apposition rate per day (Wo.B.Wi × woven bone perimeter/number of days)

In addition, the following derived variables were calculated to correct for variations in the total length of the microcrack.

1. Percentage fracture length occupied by bone formation (healing area perimeter [μm]/length of SFx [μm %])
2. Healing area per mm^2^ of the cortical bone area (healing area [μm^2^]/Ct.Ar [mm^2^])
3. Porosity BMU area per mm^2^ of the cortical bone area (porosity BMU area [μm^2^] /Ct.Ar [mm^2^])
4. Percentage fracture length occupied by erosion (erosion unhealed perimeter [μm]/length of SFx [μm %])
5. Erosion area per mm^2^ of the cortical bone area (erosion unhealed area [μm^2^]/Ct.Ar [mm^2^])

### Statistical analysis

The data collected from this experiment were analyzed using a two‐way ANOVA with time and treatment group as the independent variables. In the presence of a significant statistical interaction, the main effects in the original two‐way ANOVA were ignored, post hoc pairwise comparisons were performed between individual groups and differences determined using Fisher's least significant difference. In the presence of a nonsignificant statistical interaction, the ANOVA main effect for each independent variable (time or treatment group) was reported independently. Significance was accepted at *p* ≤ 0.05.

## Results

There were no significant differences among groups in terms of Ct.Ar and cortical bone perimeter, woven bone area (mm^2^), and the length of SFx (μm).

### Woven bone parameters

**Fig 2:**
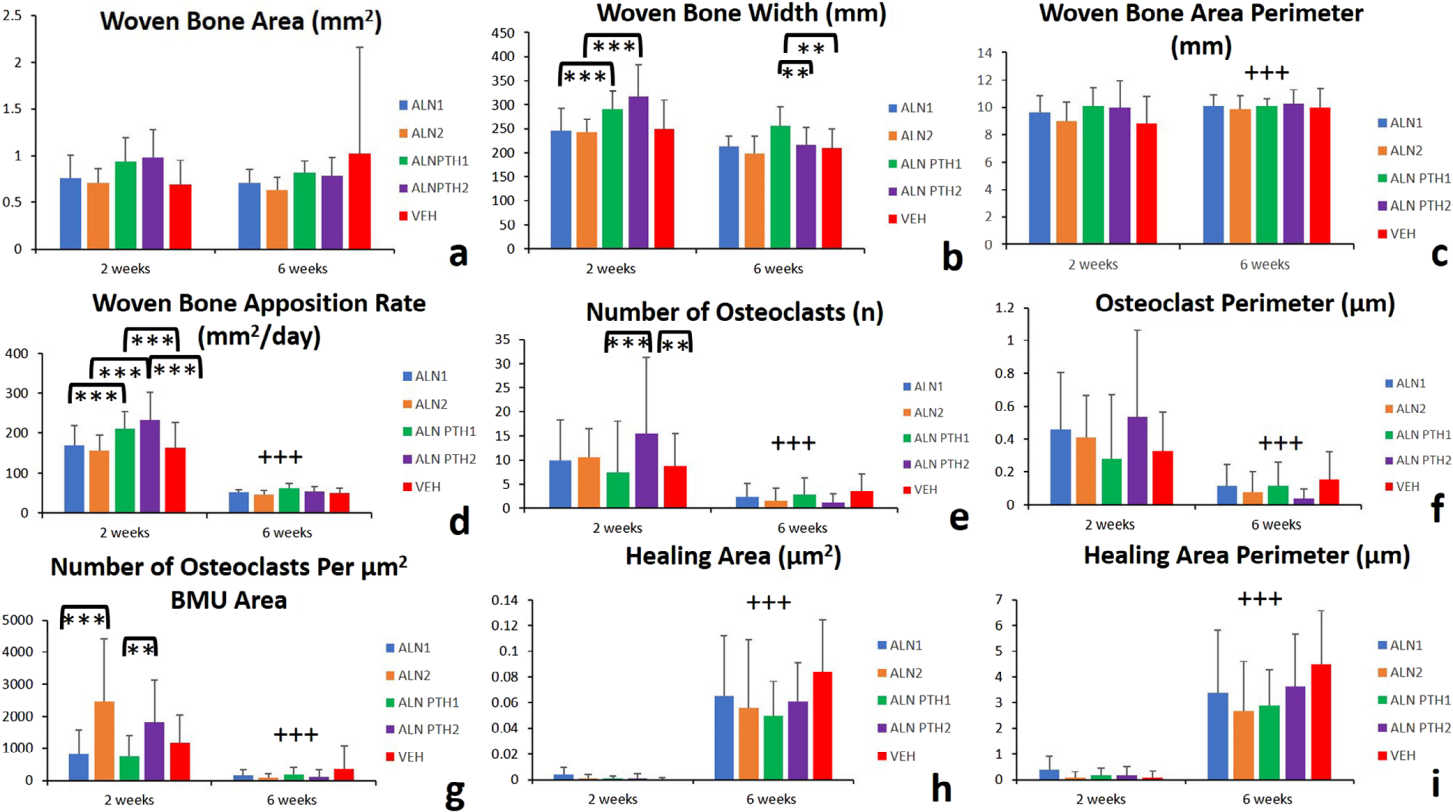
(*A–I*) Histomorphometric variables of stress fracture woven bone callus, osteoclast, and healing parameters (±SD). Combined alendronate‐parathyroid hormone (ALN‐PTH) treatment was superior to ALN and vehicle treatments in terms of woven bone sculpture and modeling throughout the healing process of a stress fracture. Cessation of ALN results in a significantly less woven bone width, indicating a more rapid progression in healing. Cessation of ALN after 2 weeks resulted in a significantly greater osteoclast number. ALN and combined ALN‐PTH treatment modes (cessation and continuation) resulted in less healing area and perimeter after 6 weeks when compared with vehicle. Most variables were significantly influenced by the main effect of time between the 2‐week and 6‐week time points. Furthermore, there was a significant interaction between the main effects of time and the treatment type on healing perimeter. ** = *p* ≤ 0.05; *** = *p* ≤ 0.01 (differences between treatment types); +++ = *p* ≤ 0.01 (differences compared with the previous time point).

There were no significant differences in woven bone area between different treatment groups (Fig. 2*A*). There was a significant interaction between the main effects of time and the type of treatment (ALN_1_ versus ALN_2_ versus ALN‐PTH_1_ versus ALN‐PTH_2_ versus VEH) on Wo.B.Wi (*F* = 3.169; *p* = 0.016). Continuation or cessation of ALN had no effect on Wo.B.Wi 2 weeks post‐SFx induction. Wo.B.Wi was significantly higher in the combined ALN‐PTH_1_ treatment group when compared with the ALN_1_ (*p* = 0.006) group after 2 weeks. It was also significantly higher in the combined ALN‐PTH_2_ treatment group when compared with the ALN_2_ (*p* < 0.001) group at the same time frame. After 6 weeks, Wo.B.Wi was significantly greater in the continuous ALN‐PTH_1_ treatment group when compared with ALN cessation in ALN‐PTH_2_ treatment (*p* = 0.05) group and the VEH group (*p* = 0.016; Fig. 2*B*).

There was no significant interaction between the effects of time and treatment type on the woven bone perimeter (Table 2). With regards to the main effect of time on woven bone perimeter, it was significantly higher at 6 weeks post‐SFx induction when compared with 2 weeks (*p* = 0.008; Fig. 2*C*).

**Table 2:** A Summary of the Significant Findings From the ALN, ALN‐PTH, and VEH Groups Related to the Effect of Time, Treatment as well as the Interaction Between Treatment and Time on Different Variables Using a Linear Model in a Two‐Way ANOVA Statistical Analysis

Variable	Effect	F	Significance
Wo.B.Wi (mm)	Treatment Time Interaction (Treatment * Time)	6.403 49.783 3.169	<0.001 <0.001 0.016
Wo.B.Pm (mm)	Treatment Time Interaction (Treatment * Time)	2.141 7.279 1.004	0.079 0.008 0.408
SFx.Po.Ar (μm^2^)	Treatment Time Interaction (Treatment * Time)	.330 31.318 .524	0.858 <0.001 0.718
SFx.Po.Pm (μm)	Treatment Time Interaction (Treatment * Time)	.556 28.886 1.010	0.695 <0.001 0.405
SFx.He.Ar (μm^2^)	Treatment Time Interaction (Treatment * Time)	.279 149.784 1.277	0.891 <0.001 0.282
SFx.He.Pm (μm)	Treatment Time Interaction (Treatment * Time)	.418 164.804 1.662	.795 <0.001 0.163
SFx.E.Ar (μm^2^)	Treatment Time Interaction (Treatment * Time)	.536 126.722 1.841	.710 <0.001 0.125
SFx.E.Pm (μm)	Treatment Time Interaction (Treatment * Time)	2.237 54.302 1.450	0.069 <0.001 0.221
N.Oc	Treatment Time Interaction (Treatment * Time)	2.431 41.695 1.847	0.05 <0.001 0.124
Oc.Pm (μm)	Treatment Time Interaction (Treatment * Time)	2.089 42.468 1.730	0.086 0.001 0.147
He%	Treatment Time Interaction (Treatment * Time)	.238 46.449 .838	0.916 <0.001 0.505
Woven bone apposition rate per day (mm^2^/day)	Treatment Time Interaction (Treatment * Time)	8.724 425.900 4.217	<0.001 <0.001 0.003
Number of osteoclasts per μm^2^ of BMU area (N.Oc/μm^2^)	Treatment Time Interaction (Treatment * Time)	6.095 46.342 4.032	<0.001 <0.001 0.005
Number of osteoclasts per μm of BMU length (N.Oc/μm)	Treatment Time Interaction (Treatment * Time)	3.559 38.909 2.816	0.009 <0.001 0.028
Healing area per mm^2^ of the cortical bone area (SFx.He.Ar [μm^2^]/ Ct.Ar [mm^2^])	Treatment Time Interaction (Treatment * Time)	.289 146.062 1.300	0.885 <0.001 0.274
Porosity BMU area per mm^2^ of the cortical bone area (SFx.Po.Ar [μm^2^] /Ct.Ar [mm^2^])	Treatment Time Interaction (Treatment * Time)	.284 153.287 .721	0.888 <0.001 0.579
Erosion (unhealed) area per mm^2^ of the cortical bone area (SFx.E.Ar, μm^2^/ Ct.Ar [mm^2^])	Treatment Time Interaction (Treatment * Time)	.503 130.923 1.946	0.733 <0.001 0.107
Percentage fracture length occupied by bone formation (SFx.He.Pm, μm/SFx.Le [μm %])	Treatment Time Interaction (Treatment * Time)	.284 153.287 .721	0.888 <0.001 0.579
Percentage fracture length occupied by erosion (SFx.E.Pm, μm /SFx.Le [μm %])	Treatment Time Interaction (Treatment * Time)	2.576 45.254 1.898	0.041 <0.001 0.115

ALN = alendronate; He% = healing percentage; N.Oc = number of osteoclasts; Oc.Pm = osteoclasts surface perimeter; SFx = stress fracture; SFx.E.Ar = erosion (unhealed) area; SFx.E.Pm = erosion (unhealed) perimeter; SFx.He.Ar = healing area; SFx.He.Pm = healing area perimeter; SFx.Le = length of stress fracture; SFx.Po.Ar = porosity BMU area; SFx.Po.Pm = porosity (BMU) area perimeter; VEH = vehicle; Wo.B.Wi = woven bone width; Wo.B.Pm = woven bone perimeter.

There was a significant interaction between the main effects of time and the type of treatment (ALN_1_ versus ALN_2_ versus ALN‐PTH_1_ versus ALN‐PTH_2_ versus VEH) on the woven bone apposition rate (*F* = 4.127; *p* = .003). The woven bone apposition rate was significantly greater in the combined treatment groups (ALN‐PTH_1_ and ALN‐PTH_2_) when compared with the ALN treatment groups (ALN_1_ and ALN_2_) after 2 weeks of SFx induction (*p* = 0.008 and *p* < 0.001, respectively). Furthermore, the woven bone apposition rate was significantly higher in the ALN‐PTH_1_ and ALN‐PTH_2_ groups when compared with the VEH group after 2 weeks of SFx induction (*p* = 0.002 and *p* < 0.001, respectively). Finally, the woven bone apposition rate decreased significantly in all groups after 6 weeks when compared with the second week (*p* < 0.001; Fig. 2*D*).

### Osteoclast parameters

There was no significant interaction between the effects of time and treatment type on the number of osteoclasts or osteoclast perimeters (Table 2).

**Fig 3:**
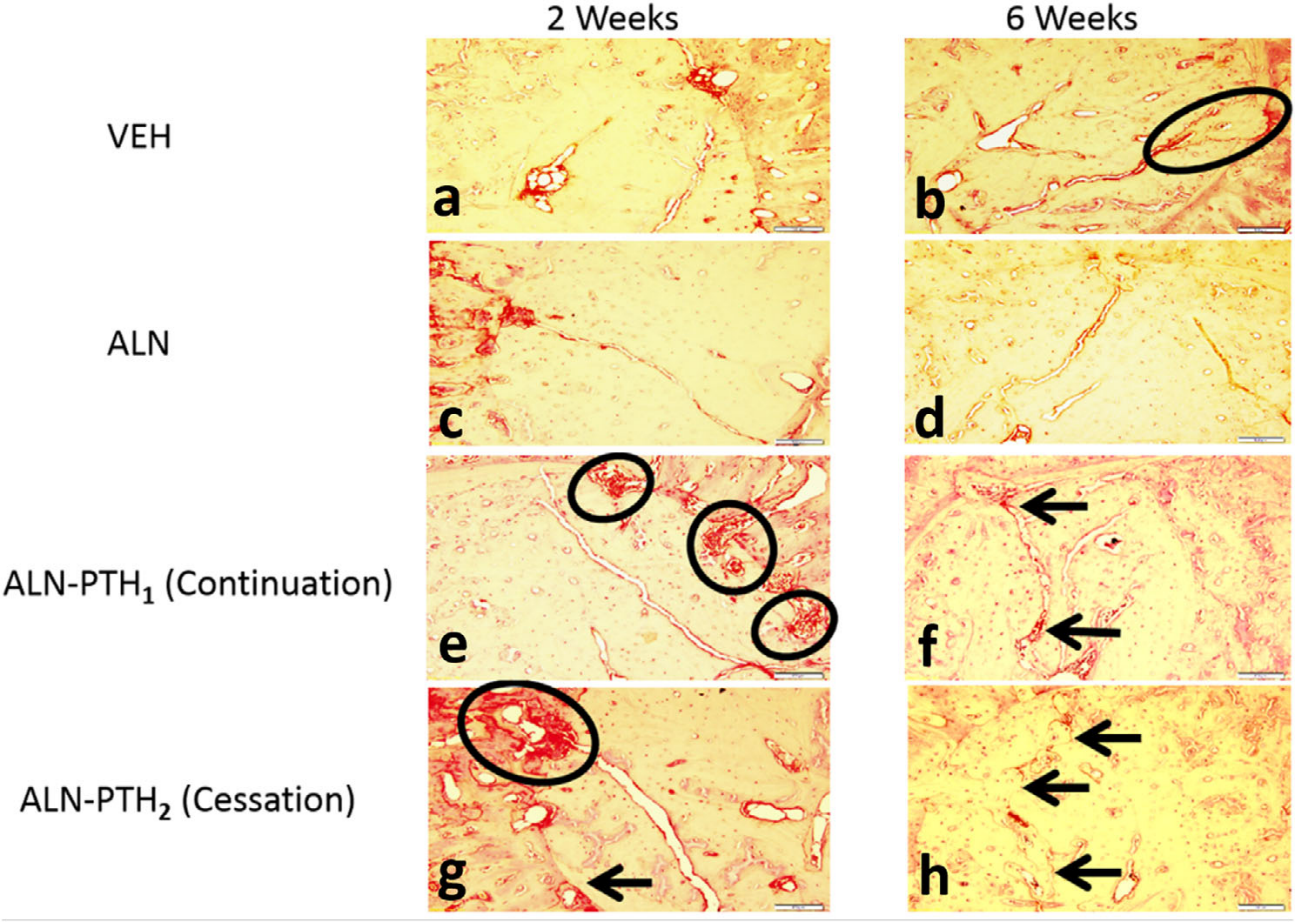
(*A–H*) Photomicrographs showing osteoclastic activity in different treatment groups and timelines. (*A,B*) Showing normal osteoclastic activity in vehicle (VEH) groups, (*C*) suppressed osteoclastic activity in alendronate (ALN) groups, and (*G*) higher osteoclastic activity in the ALN‐PTH_2_ group during the resorptive phase of the remodeling cycle after 2 weeks. (*E*) Continuation of ALN treatment in ALN‐PTH_1_ resulted in suppression of osteoclastic activity along the stress fracture despite the recruitment and availability of osteoclasts around the woven bone callus. (*B,D,F,H*) After 6 weeks, osteoclastic activity was significantly less in all groups apart from some osteoclastic trials indicating past resorptive activity. ALN‐PTH groups showed evidence of osteoclastic activity only around, but not along the stress fracture because of the progression in healing. (Tartrate‐resistant acid phosphatase 10X). Scale bar = 10 μm.

The number of osteoclasts was significantly higher in the ALN‐PTH_2_ group when compared with the ALN‐PTH_1_ (*p* = .006) and VEH groups (*p* = .024; Figs. 2*E* and 3). The number of osteoclasts and osteoclast perimeters were significantly less after 6 weeks when compared with 2 weeks post‐SFx induction (*p* < 0.001; Figs. 2*E*,*F*, and 3).

There was a significant interaction between the main effects of time and the type of treatment (ALN_1_ versus ALN_2_ versus ALN‐PTH_1_ versus ALN‐PTH_2_ versus VEH) on the number of osteoclasts per unit porosity BMU area (*F* = 4.032; *p* = .005). The number of osteoclasts per unit porosity BMU area was significantly greater after 2 weeks following cessation of ALN (ALN_2_), when compared with continuous ALN_1_ treatment (*p* < .001). Furthermore, the number of osteoclasts per unit porosity BMU area was significantly greater after 2 weeks of ALN cessation in the ALN‐PTH_2_ group when compared with when ALN treatment was continued in the ALN‐PTH_1_ group (*p* = 0.03; Figs. 2*G* and 3). With regards to the single main effect of time, the number of osteoclasts per μm^2^ of BMU area was significantly less after 6 weeks when compared with the second week in the ALN cessation groups (ALN_2_ and ALN‐PTH_2_) when compared with the ALN continuation groups (ALN_1_ and ALN‐PTH_1_; *p* < 0.001; Figs. 2*G* and 3).

There was a significant interaction between the main effects of time and the type of treatment (ALN_1_ versus ALN_2_ versus ALN‐ PTH_1_ versus ALN‐PTH_2_ versus VEH) on the number of osteoclasts per unit BMU length (*F* = 2.816; *p* = 0.028). The number of osteoclasts per unit BMU length was significantly greater 2 weeks after ceasing ALN (ALN‐PTH_2_ group) when compared with ALN continuation in the ALN‐PTH_1_ group (*p* < 0.001; Figs. 2*G* and 3). Furthermore, the number of osteoclasts per μm of BMU length was significantly less after 6 weeks post‐SFx induction when compared with the second week in the ALN_1_ group (*p* = 0.005), the ALN_2_ group (*p* = 0.001), and the ALN‐PTH_2_ group (*p* < 0.001).

### Healing parameters

**Fig 4:**
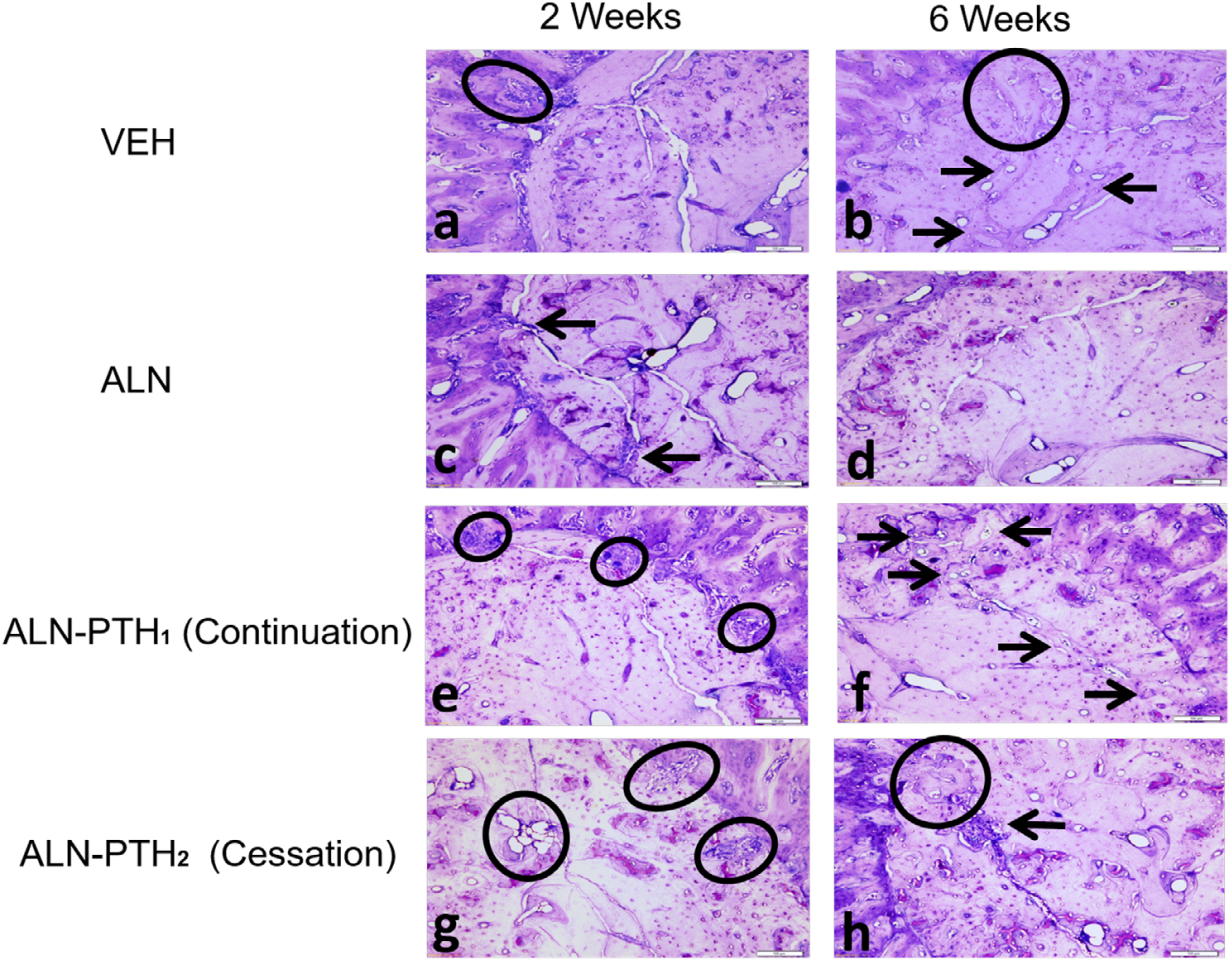
Photomicrographs showing examples of microscopic evaluation of the bone remodeling unit in stress fractures. (*A,B*) Showing the initiation of remodeling in vehicle (VEH) groups (black arrows and circles). (*C*) Alendronate (ALN) treatment resulted in the development of smaller BMUs (black arrows). (*D*) After 6 weeks, healing was completely suppressed in the ALN groups, but continued normally in the VEH groups (*B*). (*E,G*) The combined ALN‐PTH treatments triggered the development of multiple porosity areas (BMUs) that were significantly larger when ALN treatment was ceased (black circles). (*F,H*) In the combined ALN‐PTH treatment, areas of erosion (black arrows) persisted after 6 weeks along the stress fracture despite the progression of healing in other areas (black circle), especially when ALN treatment was continued (toluidine blue 10X). Scale bar = 10 μm.

**Fig 5:**
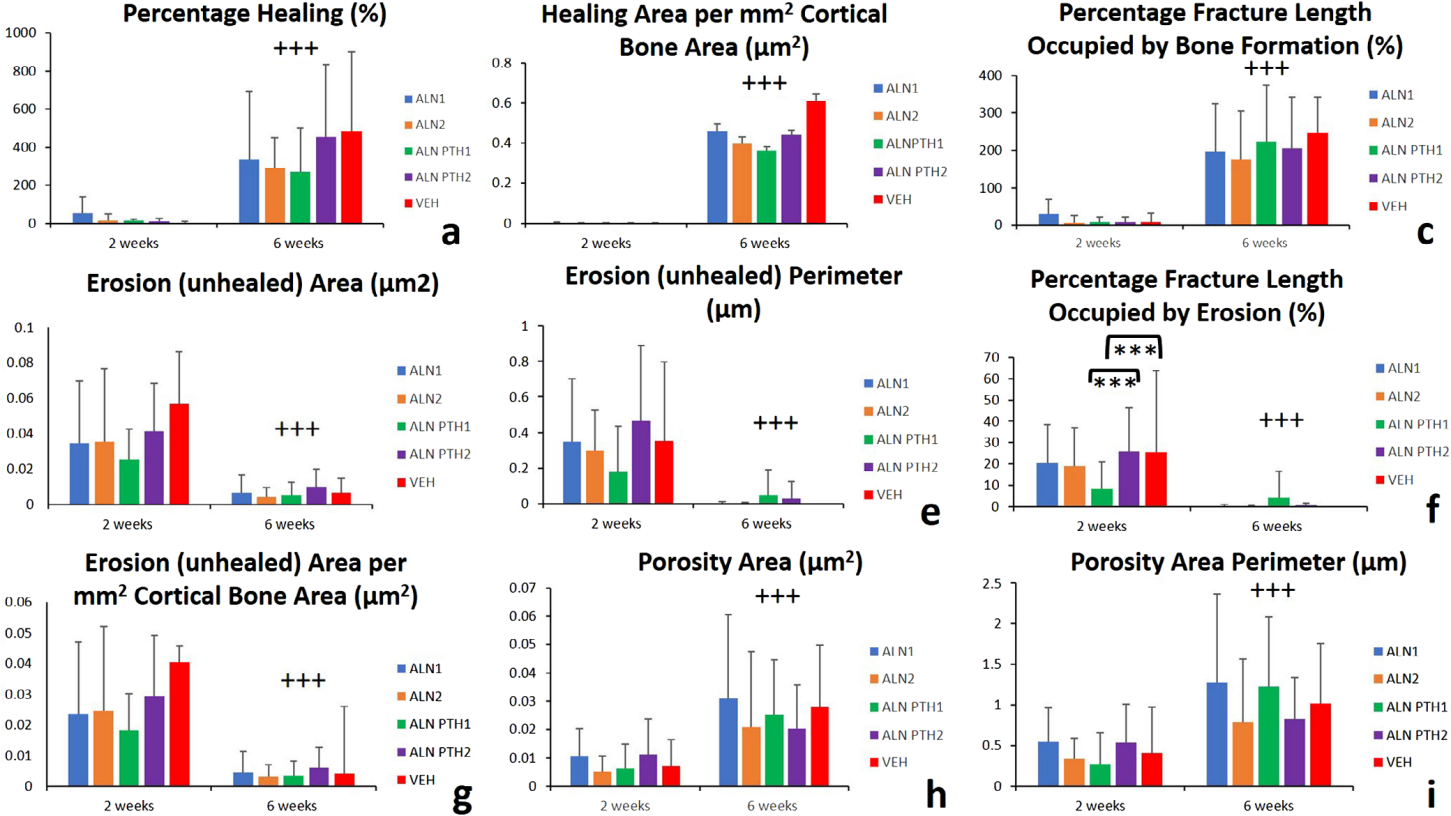
Histomorphometric variables of stress fracture healing (±SD). Continuation of alendronate in the combined alendronate‐PTH_1_ treatment mode resulted in significantly less percentage fracture length occupied by erosion after 2 weeks when compared with the vehicle and cessation of alendronate in the combined alendronate‐PTH_2_ treatment mode. Healing, porosity variables were significantly greater, whereas erosion variables were significantly less as a result of the main effect of time between the 2‐week and 6‐week time points. *** = *p* ≤ 0.01 (differences between treatment types). +++ = *p* ≤ 0.01 (differences compared with the previous time point).

There was no significant interaction between the effects of time and treatment type on any of the healing parameters (Table 2). With regards to the main effect of time, all healing parameters were significantly greater 6 weeks post‐SFx induction when compared with the second week (*p* < 0.001; Fig. 4). This finding includes the healing area (Fig. 2*H*), healing perimeter (Fig. 2*I*), healing percentage (Fig. 5*A*), healing area per mm^2^ of the Ct.Ar (Fig. 5*B*), and the percentage fracture length occupied by bone formation (Fig. 5*C*).

### Erosion (unhealed) parameters

There was no significant interaction between the effects of time and treatment type on any of the erosion parameters (Table 2).

With regards to the main effect of time, all erosion parameters were significantly less after 6 weeks of SFx induction when compared with the second week (*p* < 0.001; Fig. 5*D*,*E*,*F*,*G*). The percentage fracture length occupied by erosion was significantly less in the ALN‐PTH_1_ group, when ALN was continued, in comparison with the ALN‐PTH_2_ group, when the ALN was ceased (*p* = 0.006), and the VEH (*p* = 0.01; Fig. 5*F*) group.

### Porosity parameters

There was no significant interaction between the effects of time and treatment type on any of the porosity parameters (Table 2).

There were no significant differences in porosity parameters between the different treatment groups. With regards to the main effect of time, porosity area, perimeter, and the porosity BMU area per mm^2^ of the Ct.Ar increased significantly after 6 weeks in all treatment groups when compared with the second week (*p* < 0.001; Fig. 5*H* and *I*).

## Discussion

Our results can be explained by understanding the mechanism of healing of SFx, which occurs through direct remodeling and starts from the periosteal exit point of the SFx and progresses along the SFx line. BMUs are formed in the first 2 weeks, and osteoclasts play a significant role at this initial stage of healing.^(^ ^22^ ^)^ This is different from the repair process and healing of a complete fracture, which starts with an internal and external callus formation, with the external callus undergoing endochondral ossification for mineralization. Only after this initial phase of stabilization, bone remodeling starts to replace the lamellar bone.^(^ ^45^ ^)^ The rat model used in this study is a standardized and reliable model that has been used by us previously^(^ ^22^ ^)^; it facilitates the study of SFx remodeling. The time points chosen for histomorphometric analysis of SFx remodeling were carefully chosen in light of the fact that 2 weeks is the most representative time point for the early (resorptive) phase of the remodeling cycle, whereas 6 weeks is the most representative time point for the later (formation) phase of remodeling.

We also pretreated rats with ALN for 14 days, after which rats were treated with PTH for 14 days in the ongoing presence of ALN or its cessation. Current PTH treatment protocols are 18 to 24 months,^(^ ^46^ ^)^ which is approximately 4 weeks in a rat's life span.^(^ ^47^ ^)^ We reduced the treatment window of PTH to the shortest possible period through administration of 14 daily injections, which is sufficient to induce stable (plateau) serum PTH levels.^(^ ^48^ ^)^ The significant increase in osteoclast number along the SFx was expected at 2 weeks following PTH treatment.^(^ ^31^, ^49^, ^50^ ^)^ This may be explained by the PTH induction of monocyte chemotactic protein‐1, which is responsible for differentiation and recruitment of osteoclast precursors in early remodeling phases.^(^ ^51^, ^52^ ^)^ The mechanism of the anabolic action of PTH is also supported by other factors, including calcium availability and the calcium sensing receptor.^(^ ^53^ ^)^ The downregulation of sclerostin has also been linked to the anabolic PTH effect, through reduction of Sost expression, which negatively regulates Wnt signaling.^(^ ^54^, ^55^ ^)^

Our study is the first study to investigate the combined effect of intermittent PTH treatment with a concurrent or ceased ALN treatment on the overall remodeling of SFx. There have been inconsistent outcomes with regards to the combined antiresorptive and PTH therapies in the treatment of postmenopausal women with osteoporosis.^(^ ^32^, ^33^, ^56^, ^57^, ^58^, ^59^ ^)^ For example, greater bone turnover was achieved in women with osteoporosis receiving antiresorptive therapy (ALN) for 18 months after cessation of that therapy and switching to PTH for an additional 18 months.^(^ ^56^ ^)^ It was suggested in a previous study that the additive effect of the combined ALN‐PTH treatment is attributable to increased bone formation with a combined osteoclast inhibitory action, implying that the anabolic effect of PTH might be independent of resorption.^(^ ^60^ ^)^ Furthermore, in cases of combined treatment, decreasing the frequency of an antiresorptive agent administration results in an increase in the overall BMD.^(^ ^58^ ^)^ It was also suggested that pretreatment with PTH, followed by an antiresorptive agent like ALN and continuing the combination therapy could improve treatment outcomes.^(^ ^59^ ^)^ However, although this could be applied to chronic conditions such as osteoporosis, it is not feasible for SFx, where the timing of injury cannot be predicted beforehand to allow for sufficient time for pretreatment with PTH.

Contrary to our hypothesis, intermittent PTH treatment was more effective in SFx repair when ALN treatment was stopped. This is based on the greater recruitment and availability of osteoclasts that initiate the resorption phase of bone remodeling. Although the percentage healing of the SFx was greater following cessation of ALN, it was not significant at 6 weeks in this experiment. The current clinical treatment protocols for osteoporosis are based on long‐term drug administration with chosen periods of cessation called “drug holidays.” The evidence for the value of the drug holiday periods remains weak, and a careful evaluation for the risks versus benefits of cessation of BPs should be investigated thoroughly before making a decision.^(^ ^61^ ^)^ The optimal length of a drug holiday has not been established, but existing data suggest 3 to 5 years with ALN, 3 to 6 years with zoledronate, and 1 to 2 years with risedronate. A decision to recommence therapy should then probably be based on regular reassessment of BMD and fracture risk.^(^ ^62^, ^63^ ^)^ New alternative drugs with a shorter duration of action like denosumab^(^ ^4^ ^)^ could provide a more reversible option as an antiresorptive agent and could potentially be beneficial for patients with SFxs to prevent the risk of fractures, or at least allow for a quicker healing of an AFF caused by earlier activation of remodeling. Romosozumab (EVENITY; Amgen, Thousand Oaks, CA, USA), developed as a humanized monoclonal antibody against sclerostin,^(^ ^64^ ^)^ could also support the formation phase of remodeling.

BPs are known to have a long residence time in bone, and to also be recycled and return to other resorptive surfaces even after the treatment has ceased.^(^ ^3^, ^65^ ^)^ Limited data are available from BP discontinuation clinical trials, but suggest a residual and continuous biological effect of BPs, especially ALN and zoledronic acid for a lengthy period after discontinuation.^(^ ^66^ ^)^ It was hypothesized that BP treatment causes tissue brittleness that initiates cracks, increases homogeneity of osteonal and interstitial structures, impairs targeted repair by BMUs, and allows easier accumulation of microdamage at areas of maximum force and mechanical loading.^(^ ^67^ ^)^

In clinical studies, healing of nondisplaced AFF improved when BPs were ceased.^(^ ^68^ ^)^ There is debate as to whether the improved healing of AFF was because of teriparatide treatment, the discontinuation of BPs, or both factors combined.^(^ ^68^ ^)^ Furthermore, a reduction in the incidence of AFF by 70% per year was reported, when BPs were ceased. This was attributed to the osteoclastic bone resorption and repair of microcracks that follows the discontinuation of BP treatment.^(^ ^69^ ^)^ iPTH has shown promising results in the treatment of BP‐related AFFs.^(^ ^43^, ^68^, ^70^, ^71^, ^72^, ^73^ ^)^ In one of the case reports, despite successful healing of AFF using teriparatide, the authors questioned that it had a major role in the healing process. It was hypothesized that surgical fixation, vitamin D therapy, calcium, and ALN discontinuation could have played a secondary role in the healing process.^(^ ^10^, ^19^, ^71^, ^74^ ^)^ This aligns with the results from the current study where greater remodeling activation was observed following cessation of ALN.

In a recent case report, discontinuation of BPs and treatment with teriparatide resulted in a nearly complete radiographically verified healing of AFF, but unfortunately 12 months after the cessation of BPs, bilateral recurrence of AFF occurred, despite sequential use of teriparatide and denosumab.^(^ ^75^ ^)^ This illustrates the difficulty of managing cases of SFx in association with antiresorptive therapy. That is, cessation of antiresorptive agents is favored for healing of SFx, but compromises the structural integrity of bone in osteoporotic patients, whereas continuation of antiresorptive treatment may delay healing and lead to atypical fractures. In the absence of randomized controlled trials on this question, a taskforce of the American Society for Bone and Mineral Research recommends discontinuation of BPs, adequate calcium, and vitamin D, and consideration of teriparatide for those who appear not to heal on conservative therapy.^(^ ^15^ ^)^ The above‐mentioned results highlight the importance of the current findings related to SFx healing and note the variability of treatment outcomes as a limitation that could be related to the different animal model or unique clinical scenario of each individual case, as well as the different treatment protocols adopted in each study.

The higher values for woven bone parameters observed in this study is consistent with others,^(^ ^60^, ^76^ ^)^ and dispels the questions concerning efficacy of PTH in the formation of new bone around sites that have been previously suppressed by BP treatment.^(^ ^32^, ^33^, ^57^ ^)^ For example, PTH therapy can enable new bone formation and replace the old bone matrix on a previously resorption‐suppressed site caused by BP treatment.^(^ ^77^ ^)^ Furthermore, PTH when compared with other anabolic agents like strontium ranelate, has a long‐term stimulating effect on bone formation and resorption markers in postmenopausal women with osteoporosis previously treated with BPs.^(^ ^78^ ^)^

The present study showed that the anabolic PTH action was not affected or inhibited by prior ALN treatment. However, continuation of ALN treatment with a concurrent PTH treatment depressed its anabolic effect, and the increased Wo.B.Wi was consistent with other studies.^(^ ^60^, ^76^ ^)^ The efficacy of PTH in the formation of new bone around sites that have been previously suppressed by BP treatment had been questioned.^(^ ^32^, ^33^, ^57^ ^)^ Later, it was demonstrated that PTH could enable new bone formation and replace the old bone matrix on a previously resorption‐suppressed site following BP treatment.^(^ ^77^ ^)^ The increase in healing parameters in the ALN‐PTH group following cessation of ALN was greater, but not statistically significant, at 6 weeks. Ma and colleagues^(^ ^79^ ^)^ did show that cortical bone formation was lower in ALN‐pretreated groups when compared with PTH‐only groups. This was explained by the greater intensity of resorption during remodeling activation that leads in sequence to greater formation. The earlier increase in osteoclastic activity was certainly observed in this study.

Our results show that after 6 weeks, daily PTH injections for 14 days did not elicit the same effect, consistent with activated modeling (formation). This is in alignment with a recent study that showed a higher bone volume with higher daily PTH doses as a result of a higher trabecular number in the healing callus and a denser trabecular bone network, indicating increased bone formation.^(^ ^80^ ^)^ This opens the possibilities for investigating the effects of weekly rather than daily PTH injections, which has been investigated previously, but only in the treatment of AFF.^(^ ^73^ ^)^ It also highlights the different effects on bone surfaces, including the woven bone callus, and the remodeling required to heal the SFx line.

## Conclusion

It was concluded that cessation of ALN after SFx induction in the combined ALN‐PTH treatment was effective in increasing osteoclast parameters. Daily iPTH injections for 14 days improved the woven bone apposition rate and osteoclast parameters. Other woven bone and healing parameters remained unaffected by the cessation or continuation of ALN. It is possible that relatively short periods of iPTH therapy could activate remodeling of SFx following treatment with BPs, as long it is ceased at the time of SFx induction.

## Disclosures

The authors declare no conflicts of interest.

## AUTHOR CONTRIBUTIONS

**Mahmoud Bakr:** Conceptualization; data curation; formal analysis; investigation; methodology; project administration; validation; writing‐original draft; writing‐review and editing. **Wendy Kelly:** Data curation; investigation; project administration; writing‐review and editing. **Athena Brunt:** Data curation; investigation; methodology; project administration; writing‐review and editing. **Bradley Paterson:** Data curation; investigation; methodology; project administration. **Helen Massa:** Data curation; investigation; methodology; supervision; validation; writing‐review and editing. **Nigel Morrison:** Conceptualization; formal analysis; funding acquisition; investigation; methodology; supervision; validation; writing‐review and editing. **Mark Forwood:** Conceptualization; formal analysis; funding acquisition; investigation; methodology; project administration; supervision; validation; writing‐review and editing.
